# Getting to the heart of cardiovascular complications associated with inflammatory arthritis

**DOI:** 10.1038/s44321-025-00226-2

**Published:** 2025-04-03

**Authors:** Hong Shi, Brian H Annex

**Affiliations:** 1 Division of Rheumatology, Medical College of Georgia at Augusta University, Augusta, GA 30909 USA; 2 Division of Cardiology, Department of Medicine, Medical College of Georgia at Augusta University, Augusta, GA 30909 USA

**Keywords:** Cardiovascular System, Immunology, Respiratory System

## Abstract

Hong Shi and Brian H. Annex discuss the study by Margraf et al, in this issue of *EMBO Mol Med*, that investigates the interplay between inflammatory arthritis and cardiovascular disease, and provides a mechanistic basis for exploring FPR2-targeted therapies.

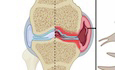

## Teaser

Inflammatory arthritis encompasses a group of autoimmune diseases, including rheumatoid arthritis (RA), psoriatic arthritis (PsA), and ankylosing spondylitis (AS) (Hammaker and Firestein, 2018). Although the exact etiology remains elusive, these conditions are thought to result from an overactive immune response to an unknown antigen, leading to joint pain, swelling, and deformity. Among these, RA is the most prevalent, affecting approximately 1% of the world’s population (Di Matteo et al, 2023). A hallmark of RA is its association with elevated rates of cardiovascular disease (CVD), which significantly increases morbidity and mortality and shortens patients’ life expectancy and quality of life (Semb et al, 2020). Although the prognosis for RA has improved markedly over the past two decades due to advanced treatment options, the lack of targeted therapies for CVD complications in RA patients remains a critical clinical challenge. Finally, guideline-based therapies for CVD are based on the etiology of the disease, which is hypertension or coronary artery disease. How well these therapies work in patients with cardiac complications of RA is incompletely understood.

In this issue of *EMBO Mol Med* (Margraf et al, 2025), a team of investigators from the laboratories of Drs. Norling and Perretti used a novel mouse model of RA that exhibits features consistent with heart failure with preserved ejection fraction (HFpEF). HFpEF is characterized by exercise intolerance, early fatigue, and dyspnea, despite normal pump/contractile function of the heart. The condition stems from impaired cardiac relaxation, which increases pressure in the pulmonary vasculature, leading to the aforementioned symptoms (Omote et al, 2022). The team’s findings provide new insights and models for cardiovascular complications in RA.

The results from Margraf A et al, demonstrate that formyl-peptide receptor (FPR) agonists alleviate cardiac dysfunction in RA. Specifically, they found that a selective FPR2 agonist effectively attenuated cardiac dysfunction and reduced joint inflammation. In contrast, a dual FPR1/FPR2 agonist failed to improve joint inflammation. Further analysis revealed common myeloid cell and fibroblast subtypes in the joints and heart that were targeted by the selective FPR2 agonist. This study provides direct evidence linking cardiac dysfunction to the inflammatory processes of peripheral arthritis. This effect was validated in human joint and cardiac fibroblasts, strengthening the translational relevance of the study.

It is well established that arthritis activity correlates with CVD risk in RA patients, with chronic inflammation serving as a primary driver of heart disease (Solomon et al, 2015). Emerging evidence suggests that persistent inflammation may result not only from excessive pro-inflammatory mediators but also from a failure to resolve pro-inflammatory responses (Chen et al, 2021). The increased FPR2 mRNA expression in RA PBMC, coupled with the distinct pro-resolving function of the selective FPR2 agonist highlighted in this study, positions FPR2 agonists as a promising therapeutic avenue for addressing CVD in RA (Lupisella et al, 2022; Chen et al, 2021).

**Figure 1:**
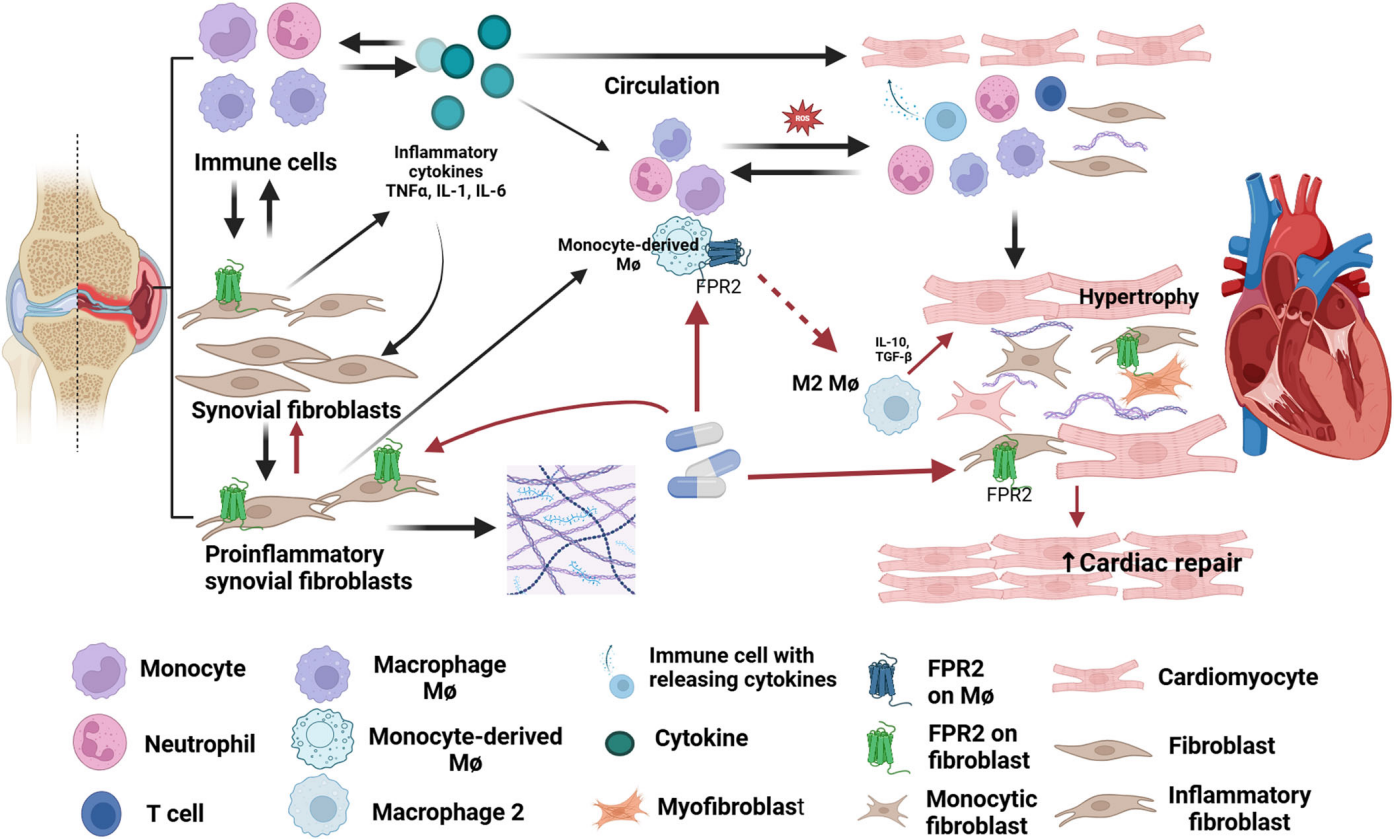
Working model for interactions of joint and cardiac inflammation in the pathogenesis of heart failure in rheumatoid arthritis. The pathogenesis of heart failure in RA patients is depicted with black lines, while the mechanisms of FPR2 agonists are illustrated with red lines. Active RA is associated with elevated circulating levels of pro-inflammatory cytokines, leading to the recruitment of leukocytes into cardiac tissues. Increased oxidative stress from infiltrating leukocytes contributes to cardiac hypertrophy. Simultaneously, active RA upregulates FPR2 expression not only in circulating myeloid cells but also in fibroblasts within both the joint and the heart. This upregulation establishes a therapeutic pathway for FPR2 agonists, highlighting their potential in targeting shared inflammatory mechanisms in RA-associated arthritis and cardiac dysfunction.

Key questions remain (Fig. 1): How exactly does joint inflammation contribute to CVD? The bone and joint microenvironment may release pro-inflammatory mediators—such as acute-phase proteins, cytokines, and chemokines—that are released and toxic to the distant heart or by promoting atherosclerosis and/or plaque rupture. Another mechanism could be the release of cells from the joint microenvironment that have the potential to promote inflammation/fibrosis. These cells could go directly to the heart or to the bone marrow for expansion. Future studies should aim to identify cytokine and/or cell mediators through comparative analyses of well-matched cohorts, including individuals with CVD but without arthritis. Advances in omics technologies using plasma and single-cell RNA sequencing may facilitate the discovery of candidate proteins or cells, assuming that relevant proteins and/or cells are freely soluble in circulation (Cuesta-López et al, 2024).

In conclusion, this study highlights the complex interplay between inflammatory arthritis and cardiovascular disease and provides a mechanistic basis for exploring FPR2-targeted therapies in RA-associated CVD. This approach may pave the way for novel, more effective treatments to improve cardiovascular outcomes in RA patients.
